# Performance of 3 Conversational Generative Artificial Intelligence Models for Computing Maximum Safe Doses of Local Anesthetics: Comparative Analysis

**DOI:** 10.2196/66796

**Published:** 2025-05-13

**Authors:** Mélanie Suppan, Pietro Elias Fubini, Alexandra Stefani, Mia Gisselbaek, Caroline Flora Samer, Georges Louis Savoldelli

**Affiliations:** 1 Division of Anaesthesiology, Department of Acute Care Medicine, Geneva University Hospitals, Rue Gabrielle-Perret-Gentil 4Geneva, 1211, Switzerland; 2 Department of Anaesthesiology, Pharmacology, Intensive Care and Emergency Medicine, Faculty of Medicine, University of Geneva, Rue Michel-Servet 1Geneva, 1211, Switzerland; 3 Division of Clinical Pharmacology and Toxicology, Department of Acute Care Medicine, Geneva University Hospitals, Rue Gabrielle-Perret-Gentil 4Geneva, 1211, Switzerland

**Keywords:** local anesthetic, dose calculation, toxicity, performance, conversational generative artificial intelligence, artificial intelligence, anesthesiology, comparative analysis, anesthetics, LA, generative artificial intelligence, ChatGPT, Copilot, Gemini, artificial intelligence models, machine learning, neural network, LLM, NLP, natural language processing, large language model, AI, ML

## Abstract

**Background:**

Generative artificial intelligence (AI) is showing great promise as a tool to optimize decision-making across various fields, including medicine. In anesthesiology, accurately calculating maximum safe doses of local anesthetics (LAs) is crucial to prevent complications such as local anesthetic systemic toxicity (LAST). Current methods for determining LA dosage are largely based on empirical guidelines and clinician experience, which can result in significant variability and dosing errors. AI models may offer a solution, by processing multiple parameters simultaneously to suggest adequate LA doses.

**Objective:**

This study aimed to evaluate the efficacy and safety of 3 generative AI models, ChatGPT (OpenAI), Copilot (Microsoft Corporation), and Gemini (Google LLC), in calculating maximum safe LA doses, with the goal of determining their potential use in clinical practice.

**Methods:**

A comparative analysis was conducted using a 51-item questionnaire designed to assess LA dose calculation across 10 simulated clinical vignettes. The responses generated by ChatGPT, Copilot, and Gemini were compared with reference doses calculated using a scientifically validated set of rules. Quantitative evaluations involved comparing AI-generated doses to these reference doses, while qualitative assessments were conducted by independent reviewers using a 5-point Likert scale.

**Results:**

All 3 AI models (Gemini, ChatGPT, and Copilot) completed the questionnaire and generated responses aligned with LA dose calculation principles, but their performance in providing safe doses varied significantly. Gemini frequently avoided proposing any specific dose, instead recommending consultation with a specialist. When it did provide dose ranges, they often exceeded safe limits by 140% (SD 103%) in cases involving mixtures. ChatGPT provided unsafe doses in 90% (9/10) of cases, exceeding safe limits by 198% (SD 196%). Copilot’s recommendations were unsafe in 67% (6/9) of cases, exceeding limits by 217% (SD 239%). Qualitative assessments rated Gemini as “fair” and both ChatGPT and Copilot as “poor.”

**Conclusions:**

Generative AI models like Gemini, ChatGPT, and Copilot currently lack the accuracy and reliability needed for safe LA dose calculation. Their poor performance suggests that they should not be used as decision-making tools for this purpose. Until more reliable AI-driven solutions are developed and validated, clinicians should rely on their expertise, experience, and a careful assessment of individual patient factors to guide LA dosing and ensure patient safety.

## Introduction

Generative artificial intelligence (AI), powered by large language models (LLMs), has emerged as a promising tool for enhancing medical decision-making [1]. These AI models, which process vast amounts of text data to generate human-like responses, have demonstrated capabilities in drug discovery and dosing optimization [2,3].

Recent studies have extensively evaluated the performance of generative AI models in medical question-answering scenarios. These models have shown promising results in medical licensing examinations [4,5] clinical case discussions and diagnostic reasoning [6,7]. However, their performance varies significantly based on task complexity. While generative AI models demonstrate strong capabilities in tasks requiring medical knowledge recall and explanation, they show limitations in scenarios demanding precise numerical calculations or complex clinical decision-making [8]. Understanding these varying capabilities of LLMs across different medical tasks is crucial when evaluating their potential role in clinical applications that require both medical knowledge interpretation and accurate numerical computations. This is particularly relevant for local anesthetic (LA) dosing, where calculation accuracy directly impacts patient safety [9,10].

LAs represent one such challenging area in clinical practice [11]. These drugs, used to induce temporary loss of sensation in specific body areas [12], require particularly careful dosing due to their narrow therapeutic window. The optimal dosing of LAs is complex, influenced by a variety of factors including patient-specific characteristics, underlying health conditions, and potential drug interactions [13].

Current methods for LA dose calculation rely heavily on empirical guidelines and clinician expertise, with no standardized recommendations universally adopted [14]. While several mobile apps exist for LA dose calculation, most allow the computation of potentially unsafe doses. Recently, LoAD Calc (Local Anesthetics Dose Calculator) was developed as a computational tool to systematize LA dose calculation [15], but like all specialized medical tools for dose calculations, it requires extensive validation to meet medical device regulations before clinical implementation. Meanwhile, health care providers increasingly turn to readily available AI models for clinical decision support [16,17]. Given this trend and the widespread accessibility of generative AI models, understanding their capabilities and limitations in LA dose calculation becomes crucial for patient safety. Empirical approaches and unsafe calculation tools can lead to overdosing and adverse outcomes, such as local anesthetic systemic toxicity (LAST) [18]. Understanding the capabilities and limitations of AI models in LA dose calculation is therefore crucial for patient safety, particularly given their widespread accessibility in health care settings [19].

In this context, generative AI emerges as a promising tool to enhance the precision of LA dose calculation. The aim of this study was to evaluate the efficacy and safety of 3 leading generative AI models in addressing the complexities of LA dose calculation. By analyzing their responses to a dedicated questionnaire including clinical vignettes, we sought to assess the accuracy and reliability of these AI algorithms in optimizing LA dosing and calculating maximum safe LA doses.

## Methods

### Study Design

This study is a comparative analysis of the performance of 3 generative AI models on the knowledge of LA dosing and computation of maximum doses in 10 simulated vignettes. Three of the most popular generative AI models: ChatGPT (OpenAI), Copilot (Microsoft Corporation), and Gemini (Google LLC), were exposed to a questionnaire about LA dose calculation once in June 2024.

### Questionnaire

A 51-item questionnaire, derived from a protocol developed by anesthesiologists to test LA calculation by clinicians [20], included 3 questions on model performance in answering medical questions and output accuracy, 17 questions on LA dose calculation specifics, 1 introductory question on dose determination in clinical vignettes, and 10 clinical vignettes, each followed by 2 questions on the assessed safety of model outputs. These clinical vignettes were initially created to carry out a parallel group randomized controlled trial, the protocol of which has already been published [20]. The purpose of these vignettes was to compute the maximum safe dose of 3 commonly used LAs, alone or in combination (mixture of 2 different LAs). Different clinical settings were described, and the patients’ physical characteristics, comorbidities, and medications varied significantly. The complete questionnaire is available in Multimedia Appendix 1.

### AI Model Data Generation

We analyzed the latest stable versions of 3 generative AI models, namely ChatGPT-4.0, Microsoft Copilot, and Google Gemini 1.0. These models were selected due to their popularity at the time of the study, their widespread accessibility in health care settings, and their representation of current state-of-the-art technology from 3 leading AI companies (OpenAI, Microsoft, and Google) [21,22]. All models were accessed through their public web interfaces using standard parameter settings between noon and 5:00 PM UTC during our data collection period (June 19‐24, 2024). Each model was presented with the exact prompts provided in the questionnaire (Multimedia Appendix 1) in a standardized sequence. Given the stochastic nature of LLMs, which can produce varying responses across multiple runs, we opted for a single-run approach to mirror real-world clinical scenarios where practitioners typically rely on single queries. The responses were recorded in a separate Microsoft Word file for subsequent analysis.

### Definition of Maximum Safe Doses

The expected maximum safe doses were determined manually using a set of scientifically grounded calculation rules previously described and used in the development of the LoAD Calc app [15]. The anticipated results were calculated using the app itself. Before the study, these results were cross-checked by 3 anesthesiologists who manually recomputed the calculations for each vignette using the LoAD Calc calculation rules, without using the app itself.

Typically, maximum safe doses are calculated in milligrams. However, in clinical practice, anesthesiologists administer a volume of LA, the concentration of which can vary, rather than a specific quantity of LA. Thus, while toxicity correlates with the quantity (in milligrams) of LA administered, it is more clinically relevant to determine the maximum volume (in milliliters) of LA suitable for a particular patient and a specific LA concentration. Therefore, half of the vignettes required calculating volumes while the other half dealt with milligrams.

Initially, each maximum safe dose was calculated in milligrams and then converted back to milliliters based on the concentration of the LA used in the vignette. This volume was rounded down to the nearest integer. An overdose was defined as any dose exceeding this maximum volume or its corresponding quantity of LA in milligrams.

### Quantitative Evaluation

For the quantitative evaluation values in milligrams or milliliters given by each AI model were compared with the values computed with the full set of rules. Briefly, the first step was to determine the calculation weight (CW). To determine the CW, the BMI and ideal body weight (IBW) using Devine formula were calculated [23]. CW was capped at 70kg to ensure safe LA doses. The CW was determined as follows:

1. If actual weight (AW) was ≤70 kg, BMI<30 kg/m², and IBW >AW, then CW=AW.
2. If AW≤70 kg, BMI<30 kg/m², and IBW≤AW, then CW=IBW.
3. If AW≤70 kg and BMI≥30 kg/m², then CW=IBW.
4. If AW>70 kg and IBW>70 kg, then CW=70 kg.
5. If AW>70 kg and IBW≤70 kg, then CW=IBW.

Next, the maximum safe dose was adjusted based on patient factors affecting LA metabolism. For patients aged 70 years or older, with renal dysfunction (glomerular filtration rate <50 mL/min), hepatic dysfunction (prothrombin time <50%), heart failure (left ventricular ejection fraction ≤30%), pregnancy, or using major cytochrome P450 1A2 or 3A inhibitors (eg, ciprofloxacin and macrolides), the maximum dose was reduced by 20%. If 2 or more of these factors were present, it was reduced by 30%. A simplified calculation relies solely on the patient’s AW or IBW to compute the maximum safe dose using the following formula [24]:


$$Maximum\;dose\left(mg\right)=Weight\left(AW\vee IBW\right)\left(kg\right)\times Dose\;limit\;for\;chosen\;LA\left(\frac{mg}{kg}\right)$$

While patient-specific adaptations are important for safety, a simplified calculation method relying solely on patient weight is more commonly used in clinical practice [19]. This dual approach reflects the complexity of LA dosing, where multiple calculation methods coexist. While we chose the comprehensive method as our primary evaluation criteria for its rigorous safety assessment, including the simplified method as a secondary outcome helps contextualize our findings within current clinical practices.

### Qualitative Evaluation

To conduct a qualitative assessment, a comprehensive list of elements crucial for reproducing the calculation rules used by LoAD Calc was predefined. From this selection, a detailed list of items was compiled and organized in a Microsoft Excel file (Multimedia Appendix 2). The 2 independent reviewers were board-certified anesthesiologists with over 5 years of clinical experience in regional anesthesia and LA dose calculation. Their familiarity with LoAD Calc in both clinical practice and research settings ensured a thorough understanding of LA dosing principles. For each element, reviewers evaluated domains by considering the accuracy of dose calculations compared with reference values, consistency between stated principles and computed doses, and relevance of provided explanations to clinical practice. These aspects were synthesized into a single rating for each domain. This balanced approach aimed to evaluate the performance of AI models beyond just numerical accuracy. The reviewers, blinded to the AI models, assessed the performance of each AI for every predefined individual item on a 5-point Likert scale (1=very poor, 2=poor, 3=fair, 4=good, and 5=very good).

### Outcomes

The primary outcome was the overall overdose rate using the comprehensive set of calculation rules used in the development of LoAD Calc. The secondary outcomes included assessing the overdose rate based on the simulated patient’s IBW and AW, as well as examining the overdose rate associated with each studied LA. In addition, a qualitative evaluation was conducted to gauge the AI’s proficiency in considering individual elements of the calculation process.

### Statistical Analysis

Data were entered in an Excel Binary File Format (.xls) file and curated using Stata (version 17.0; StataCorp LLC). If ranges were suggested by the AI model, the lowest dose advised was used. Descriptive characteristics were reported using means and SDs, as were the LA values exceeding the reference doses. The frequencies of categorical variables were calculated and reported in percentages. An overall value was computed for the qualitative evaluation of each AI model and rounded to the nearest integer to report a consistent rating. Each element was also specifically analyzed, and ratings reported accordingly. When reviewers disagreed on a rating, the median value was computed and rounded to the nearest integer. Cronbach α coefficient was computed to assess inter-rater reliability.

## Results

All 3 models were able to complete the questionnaire. The complete questionnaires with the answers given by each model can be found in Multimedia Appendix 3.

Gemini only generated 3 ranges of values (3/10, 30%), one for each LA tested. In the 2 instances where mixtures were used (2/3, 67%), the values provided exceeded maximum safe doses by 140% (SD 103%) (Table 1). This model’s responses contained no precise dose calculations or specific doses or volumes, and included statements about consulting medical professionals for accurate dosing guidance.

**Table 1.** Detailed values provided by Gemini, ChatGPT, and Copilot for each vignette. The maximum safe doses were computed using the full calculation rules.

Vignette	Local anesthetic		Reference value	Mixture	Gemini	ChatGPT	Copilot
1	Ropivacaine (mg)		165	No	150	165	165
2	Levobupivacaine (mL)		9	Yes	15	20.6	10
3	Lidocaine (mg)		40	Yes	—^a^	270	270
4	Levobupivacaine (mL)		18	No	—	21	28
5	Levobupivacaine (mg)		110	No	—	240	240
6	Ropivacaine (mL)		18	Yes	—	64	—
7	Levobupivacaine (mg)		82.5	No	—	120	150
8	Lidocaine (mL)		6	Yes	18.75	33.75	33.75
9	Ropivacaine (mg)		123.75	No	—	270	33.75
10	Ropivacaine (mL)		29	No	—	48	16

a Not available.

ChatGPT provided values for all vignettes. These values were unsafe in 9 cases (9/10, 90%). In unsafe cases, the values proposed by the AI model exceeded maximum safe doses by 198% (SD 196%; 129, SD 143 mg for lidocaine, 46, SD 58 mg for levobupivacaine, and 70, SD 67 mg for ropivacaine).

Copilot provided values for 9 cases (9/10, 90%). These values were always safe when ropivacaine was the LA tested. They were nevertheless unsafe in the 6 cases where either lidocaine or levobupivacaine were used (6/9, 67%). In these cases, the values proposed by the AI model exceeded maximum safe doses by 217% (SD 239%; 129, SD 143 mg for lidocaine and 52, SD 60 mg for levobupivacaine). When values lower than the ones used as reference were given no details on the calculation were given. Detailed values are given in Table 1.

When considering IBW, the proportion of LA overdose remained unchanged with Gemini. It was of 70% (7/10) with ChatGPT and 56% (5/9) with Copilot. When the patient’s actual weight was the only parameter taken into account to determine maximum LA doses, the values provided by Gemini were still too high in the 2 instances where mixtures were used (2/3, 67%). However, the proportion of LA overdose dropped to 40% with ChatGPT, and to 33% with Copilot.

The qualitative assessments conducted by the 2 independent reviewers showed high consistency, with a Cronbach α value of 0.87 and no differences exceeding a single level on the Likert scale. A total of 5 disagreements were recorded for Gemini and Copilot (5/8, 63%), and only 1 for ChatGPT (1/8, 13%). Gemini was rated as “fair,” while both ChatGPT and Copilot were rated “poor.” Copilot had the highest rate of “very poor” ratings (3/8, 38%; Table 2).

**Table 2.** Qualitative analysis of Gemini, ChatGPT, and Copilot for specific local anesthetics dose calculation elements.

Criteria for dose adaptation	Gemini	ChatGPT	Copilot
Height and weight	Poor	Poor	Poor
Age	Fair	Poor	Poor
Renal dysfunction	Good	Good	Fair
Hepatic insufficiency	Fair	Poor	Fair
Heart failure	Fair	Poor	Poor
Pregnancy	Fair	Very poor	Very poor
Drugs decreasing LA^a^ metabolism	Fair	Poor	Very poor
Use of LA mixtures	Poor	Poor	Very poor
Overall	Fair	Poor	Poor

a LA: local anesthetic.

## Discussion

### Principal Findings

In this study, the evaluated generative AI models generally advised unsafe LA doses when confronted with realistic clinical vignettes. The analysis showed considerable variability in the outputs from these models, with Gemini’s responses containing the fewest unsafe doses but also providing the least number of specific recommendations. Importantly, most AI-generated doses were deemed unsafe when evaluated against a comprehensive set of calculation rules that prioritize the lowest, safest dose. Even when using less stringent criteria, AI models still tended to recommend excessively high doses, raising serious safety concerns about their potential use in clinical practice.

While our study focused on general-purpose AI models, it’s worth noting that specialized tools for LA dose calculation are rare. As analyzed in our previous work [15], most available tools for LA dose calculation were found to be potentially unsafe, allowing computation of excessive doses. LoAD Calc, which served as our reference standard, was specifically designed to address these safety concerns. The significant performance gap between this purpose-built medical tool and general-purpose AI models highlights the importance of domain-specific knowledge and safety constraints in clinical applications.

While the models’ responses included general recommendations about dose adaptation based on patients’ comorbidities or treatments, when asked to perform specific calculations in the clinical vignettes, the calculated outputs showed significant inconsistencies. The limitations in dose adaptation calculations based on patient-specific factors, such as comorbidities or drug interactions, further underscore their limitations [8]. Personalized medicine requires an approach that AI models currently cannot provide adequately [10]. Furthermore, all models underperformed when tasked with calculating doses for LA mixtures, a common practice in anesthesiology, indicating their current inadequacies in complex clinical scenarios [25].

These findings align with previous research that has questioned the reliability of AI in critical medical applications. For instance, while AI has demonstrated promise in diagnostic imaging and drug discovery, its performance in decision-making tasks like diagnosis and dose calculation, remains inconsistent [26]. When processing multiple clinical variables, these models generate errors that can compromise patient safety [27,28].

The clinical implications are especially concerning given the severe consequences that can arise from LA dosing errors. When safe dosing limits are exceeded, LAST can manifest through central nervous system toxicity (seizures and loss of consciousness) and cardiovascular collapse. This is especially alarming for the high-risk scenarios in our vignettes involving patients with organ system dysfunction (hepatic, renal, or cardiac), advanced age, or concurrent medications affecting LA metabolism, where the safety margin is already reduced. The significant overdosing we observed with LA mixtures is particularly dangerous in clinical practice, as the combined toxicity of multiple agents can potentiate adverse effects and complicate resuscitation efforts if LAST occurs.

A notable concern was the lack of transparency in how AI models like Copilot arrived at their dose recommendations, sometimes suggesting lower, safe doses without clear explanations. This “black box” nature poses significant risks, as it prevents users from understanding the AI’s decision-making process, potentially leading to errors [29]. In addition, AI models are susceptible to hallucinations, generating content that is not based on real or existing data and thus misrepresenting reality [30]. Previous research has also demonstrated that generative AI can fabricate references, misleading users into believing that the information provided is scientifically grounded [31].

In the qualitative assessment, Gemini received the highest overall rating for its explanations on adjusting doses according to different patient characteristics and medications. However, its tendency to withhold exact dosage recommendations, opting for a safer approach, diminishes its usefulness for dose computation. While this conservative approach of recommending specialist consultation aligns with safety principles, it limits practicality for real-time clinical use. As noted in previous research, optimizing Gemini to provide more direct answers to medical queries could enhance its use [32].

Given these outcomes, the applicability of these 3 generative AI models in clinical practice for LA dose calculation remains limited. The AI models tested were unable to consistently provide safe and accurate dosage recommendations, which is crucial in anesthesiology to prevent complications such as LAST. Health care professionals should exercise caution when considering the use of generative AI models for LA dose calculation. The study suggests that while AI has potential in certain aspects of medical practice, its application in dose computation for LAs is potentially dangerous and therefore premature. Until AI models can reliably incorporate complex, patient-specific factors and adhere to stringent safety guidelines, their role should be limited to supplementary tools rather than primary decision-makers [33,34]. Preference should be given to AI systems that are transparent in their decision-making processes, allowing clinicians to understand and verify recommendations. AI models that integrate continuous learning capabilities and up-to-date medical guidelines would be more suitable for clinical applications. At present, clinicians should prioritize their clinical judgment and experience, carefully evaluating individual patient factors to guide LA dosing and maintain patient safety.

This study evaluated generative AI models through their default public interfaces using standardized prompting, mirroring how health care providers would typically access these tools in clinical practice. This methodological choice was deliberate, as surveys indicate most clinicians use these models through standard web interfaces rather than fine-tuned versions or specialized prompting strategies [16,19]. While fine-tuning these models with domain-specific data on LA dosing might potentially improve performance, such customization requires technical expertise, computational resources, and access to proprietary APIs, resources generally unavailable to most health care providers. In addition, even fine-tuned models would require rigorous clinical validation equivalent to medical devices before implementation in clinical practice, highlighting the gap between technological capability and clinical applicability.

Our standardized prompting approach focused on obtaining direct dosing recommendations rather than explicitly requesting step-by-step reasoning processes, aligning with our primary research objective of evaluating output safety and reliability rather than reasoning transparency. As previous studies have noted, generative AI models can demonstrate a disconnect between reasoning and output accuracy, providing seemingly sound explanations for incorrect outputs or correct answers with flawed reasoning [6].

### Limitations

This study has several limitations. The evaluation was based on simulated clinical vignettes, which, while designed to mimic real-world scenarios, cannot capture the full complexity of actual clinical practice. While our set of vignettes was designed to cover major clinical variables affecting LA dosing, we recognize that real-world scenarios present an even wider range of patient characteristics and clinical contexts. In addition, the study relied on predefined calculation rules and expert evaluations, which, while rigorous, may not encompass all possible clinical scenarios or dosing variations. Furthermore, the study focused on 3 specific AI models, currently available to the public, so the findings may not be generalizable to other generative AI systems or future iterations of these models. Another limitation is the static nature of AI models, which lack the ability to update their knowledge or reasoning processes in real-time. This is particularly problematic in medicine, where new research and clinical guidelines continually evolve. Without regular updates to their training data, AI models may quickly become outdated, leading to recommendations that do not reflect current best practices. Finally, our single-run methodology, while reflecting typical clinical usage where practitioners rely on single queries, presents a limitation given the stochastic nature of LLMs. This methodological choice prevents assessment of response consistency and reliability across multiple attempts, particularly relevant for drug dosing calculations, where response variability could have safety implications. Future research could explore whether fine-tuned models specifically trained on LA dosing guidelines or alternative prompting strategies requesting step-by-step reasoning might improve calculation accuracy. In addition, multiple runs should be considered to evaluate response consistency and establish confidence intervals for dosing recommendations. Such investigations could enhance our understanding of these models’ limitations while maintaining the focus on patient safety.

### Conclusion

In conclusion, while generative AI models like Gemini, ChatGPT, and Copilot offer significant promise, their current capabilities fall short in the critical area of LA dose calculation. The study’s findings suggest that these AI tools are not yet ready for clinical use in this context, primarily due to their inconsistent performance and the potential for recommending unsafe dosages. Future advancements in AI technology must focus on enhancing the accuracy, transparency, and adaptability of these models to ensure they can be safely integrated into medical practice. Until then, reliance on clinician expertise and established dosing tools remains essential for ensuring patient safety.

## Supplementary material

10.2196/66796 Multimedia Appendix 1 Local anesthetic dose calculation questionnaire.

10.2196/66796 Multimedia Appendix 2 Artificial intelligence model qualitative evaluation sheet.

10.2196/66796 Multimedia Appendix 3 Responses from artificial intelligence models on local anesthetic dose calculation.

## Abbreviations

AI: artificial intelligence
AW: actual weight
CW: calculation weight
IBW: ideal body weight
LA: local anesthetic
LAST: local anesthetic systemic toxicity
LLM: large language model
LoAD Calc: Local Anesthetics Dose Calculator
