# Supplementary material for: Performance of 3 Conversational Generative Artificial Intelligence Models for Computing Maximum Safe Doses of Local Anesthetics: Comparative Analysis
Source: JMIR AI. 2025 May 13;4:e66796. doi: 10.2196/66796 (PMC12223683; doi:10.2196/66796)
Supplement: Multimedia Appendix 1 [file ai-v4-e66796-s001.docx]

**Questionnaire for conversational generative AI**

**(ChatGPT / Copilot / Gemini)**

Can you answer professional medical questions?

Is there a subscription version which would give more accurate or relevant answers?

How confident should I be in the answers you will provide me with?

Can you calculate maximum safe injectable doses of local anesthetics?

What is the maximum safe dose of bupivacaine that can be injected for a peripheral nerve block?

What is the maximum safe dose of bupivacaine that can be injected intravenously?

What is the maximum safe dose of levobupivacaine that can be injected for a peripheral nerve block?

What is the maximum safe dose of levobupivacaine that can be injected intravenously?

What is the maximum safe dose of lidocaine that can be injected for a peripheral nerve block?

What is the maximum safe dose of lidocaine that can be injected intravenously?

What is the maximum safe dose of ropivacaine that can be injected for a peripheral nerve block?

What is the maximum safe dose of ropivacaine that can be injected intravenously?

Can you convert a local anesthetic dose given in milligrams into a volume in milliliters if the concentration is known?

Can you do the reverse operation, i.e. convert a volume of local anesthetic in milliliters into a dose in milligrams if the concentration is known?

Can you determine the maximum dose of local anesthetics when a mixture of 2 different local anesthetics is used?

Do you know if it's necessary to adapt local anesthetic doses to the patient's weight and height?

Do you know if it's necessary to adapt local anesthetic doses according to the patient's age?

Do you know which comorbidities require adaptation of local anesthetic doses?

Do you know whether taking certain medications requires adaptation of local anesthetic doses?

Would you know how to adapt these doses to a particular patient, taking into account weight, height, age, comorbidities and current medications?

Would you be able to determine maximum doses of local anesthetics taking into account patient specifics for several clinical vignettes?

Vignette 1: 33-year-old man (55kg for 174cm), ASA 2, active smoker (15 UPA), in usual good health except undernutrition. Liver and renal function are normal. He has no cardiac or respiratory comorbidities. The patient does not take any medication. He is scheduled for ligament surgery of the left shoulder. In agreement with the patient, you opt for an interscalene block followed by general anesthesia. What is the maximum dose (in milligrams) of ropivacaine 0.5% that I could inject to perform this block on this patient?

So, I can safely administer this dose to the patient I described, right?

And there would be no risk of local anesthetic systemic toxicity with this dose?

Vignette 2: 74-year-old woman (51.5kg for 159cm), ASA 2, known for hypertension, gastroesophageal reflux and hypercholesterolemia. Liver and renal function are normal. She has no other cardiac or respiratory comorbidities. Her usual treatment consists of CoLisinopril (lisinopril and hydrochlorothiazide) and atorvastatin. She is scheduled for a total left knee replacement. The anesthetic strategy chosen, in agreement with the patient, was a femoral block (with ropivacaine 0.5%) and a popliteal plexus block (with levobupivacaine 0.5%) prior to general anesthesia. You have already performed the femoral block using 10 ml ropivacaine 0.5%. What is the maximum volume (in milliliters) of levobupivacaine 0.5% that you could inject to perform the popliteal plexus block in this patient?

So, I can safely administer this dose to the patient I described, right?

And there would be no risk of local anesthetic systemic toxicity with this dose?

Vignette 3: 32-year-old woman (60kg for 170cm), ASA 2 for first harmonious pregnancy currently at 30 weeks' amenorrhea. Liver and renal function are normal. She has no cardiac or respiratory comorbidities. She doesn’t take any medication. She is scheduled for osteosynthesis of the left wrist following a fracture of the distal end of the radius. Taking the clinical context into account and in agreement with the patient, you opt for an axillary block. The nurse has already opened 2 vials of 10ml ropivacaine 0.5% which you decide to use entirely. You would also like to add lidocaine 1% so that you can quickly check the effectiveness of the block. What is the maximum dose (in milligrams) of lidocaine 1% that you could inject (in addition to the 20ml of ropivacaine 0.5%) to perform this block on this patient?

So, I can safely administer this dose to the patient I described, right?

And there would be no risk of local anesthetic systemic toxicity with this dose?

Vignette 4: 75-year-old man (70kg for 170cm), ASA 2, known for hypertension and chronic renal failure with an estimated glomerular filtration rate (eGFR) of 30 ml/min. His liver function tests are normal. He has no cardiac or respiratory comorbidities. His usual treatment includes lisinopril, amlodipine and torasemide. He is scheduled for arthroscopy of the left knee. In agreement with the patient, you opt for a saphenous nerve block (with 0.5% levobupivacaine) prior to general anesthesia. What is the maximum volume (in milliliters) of levobupivacaine 0.5% that you could inject to perform this block on this patient?

So, I can safely administer this dose to the patient I described, right?

And there would be no risk of local anesthetic systemic toxicity with this dose?

Vignette 5: 37-year-old woman (120kg for 180cm), ASA 2, known for hypertension and obesity. Liver and renal function are normal. She has no other cardiac or respiratory comorbidities. She is recovering from a lower urinary tract infection treated with ciprofloxacin (with the last scheduled dose today). She only takes enalapril 10mg once daily. She is scheduled for stabilization of an ankle fracture. In agreement with the patient, you decide to perform a popliteal sciatic block (levobupivacaine 0.5%) prior to general anesthesia. What is the maximum dose (in milligrams) of levobupivacaine 0.5% that you could inject to perform this block on this patient?

So, I can safely administer this dose to the patient I described, right?

And there would be no risk of local anesthetic systemic toxicity with this dose?

Vignette 6: 65-year-old woman (80kg for 175cm), ASA 3, known for GOLD 2 chronic obstructive pulmonary disease and Child B hepatic cirrhosis consecutive to nonalcoholic steatohepatitis (bilirubin 40 µmol/l, albumin 30 g/l, PT 45%, thrombocytes 150 G/l, no ascites or encephalopathy), hypercholesterolemia and noninsulin-requiring type 2 diabetes. A hemostasis consultation found no contraindication to locoregional anesthesia, the disturbance in PT being due solely to the progression of liver disease. Renal function tests are normal. The patient has no cardiac comorbidities. Her usual treatment includes atorvastatin, metformin and Spiriva. She is scheduled for a total left knee replacement. In agreement with the patient, you opt for a femoral block (with 12ml of levobupivacaine 0.5%) and a popliteal plexus block (with ropivacaine 0.375%) prior to general anesthesia. You have already performed the femoral block using 12 ml of levobupivacaine 0.5%. What is the maximum volume (in milliliters) of ropivacaine 0.375% that you could inject to perform the popliteal plexus block in this patient?

So, I can safely administer this dose to the patient I described, right?

And there would be no risk of local anesthetic systemic toxicity with this dose?

Vignette 7: 80-year-old man (60kg for 175cm), ASA 3, known for paroxysmal atrial fibrillation, who underwent TAVI (Transcatheter Aortic Valve Implantation) 1 year ago for severe aortic stenosis with left ventricular ejection fraction at 30%. He is also known for a prior left sylvian stroke (no sequelae), hypertension and hypercholesterolemia. He is active and independent for activities of daily living. Liver and renal function are normal. His usual treatment includes Co-Lisinopril (lisinopril and hydrochlorothiazide), atorvastatin and rivaroxaban, with the latter currently on hold. He is scheduled for a cephalic left hip replacement. In agreement with the patient (who categorically refuses neuraxial anesthesia), you opt for a femoral and lateral thigh cutaneous nerve block (with levobupivacaine 0.375%) prior to general anesthesia. What is the maximum dose (in milligrams) of levobupivacaine 0.375% that you could inject to perform this block on this patient?

So, I can safely administer this dose to the patient I described, right?

And there would be no risk of local anesthetic systemic toxicity with this dose?

Vignette 8: 57-year-old woman (75kg for 170cm), ASA 1, in usual good health. Liver and renal function are normal. She has no cardiac or respiratory comorbidities. She does not take any medication. She is scheduled for osteosynthesis of the right ankle. In agreement with the patient, you opt for a popliteal sciatic block (with levobupivacaine 0.5%) and a saphenous block (with lidocaine 1%). You have already performed the popliteal sciatic block by injecting 15ml of levobupivacaine. What is the maximum volume (in milliliters) of 1% lidocaine you could inject to perform the saphenous nerve block on this patient?

So, I can safely administer this dose to the patient I described, right?

And there would be no risk of local anesthetic systemic toxicity with this dose?

Vignette 9: 84-year-old woman (90kg for 160cm), ASA 2, known for hypertension and obesity. Her hypertension is currently untreated due to hypotensive episodes with falls under the previous anti-hypertensive. Liver and renal function are normal. She has no other cardiac or respiratory comorbidities. She is scheduled for a right shoulder prosthesis. In agreement with the patient, you opt for an interscalene block (with ropivacaine 0.375%) followed by general anesthesia. What is the maximum dose (in milligrams) of ropivacaine 0.375% that you could inject to perform this block on this patient?

So, I can safely administer this dose to the patient I described, right?

And there would be no risk of local anesthetic systemic toxicity with this dose?

Vignette 10: 35-year-old woman (80kg for 157cm), ASA 2, known to have type 1 diabetes on insulin pump. She has no cardiac or respiratory comorbidities. Liver and renal function are normal. She is scheduled for foot surgery. In agreement with her, you opt for an ankle block (with ropivacaine 0.5%). What is the maximum volume (in milliliters) of ropivacaine 0.5% that you could inject to perform this block on this patient?

So, I can safely administer this dose to the patient I described, right?

And there would be no risk of local anesthetic systemic toxicity with this dose?
